# Citizen consensus and diverging views on benefit-sharing for genetic resources

**DOI:** 10.1038/s44185-025-00093-7

**Published:** 2025-07-03

**Authors:** Anna Lou Abatayo, Xiaolongren Ding, Esteban Neira-Monsalve, Andries Richter

**Affiliations:** 1 Environmental Economics and Natural Resources Group, Wageningen University and Research, Wageningen, 6706KN the Netherlands; 2 Business Economics Group, Wageningen University and Research, Wageningen, 6706 KN the Netherlands; 3 Bioprocess Engineering Group, Wageningen University and Research, Wageningen, 6706KN the Netherlands

**Keywords:** Society, Decision making

## Abstract

The governance of genetic resources and their digital sequence information (DSI) faces challenges in achieving globally equitable benefit-sharing under the Convention on Biological Diversity. Citizens in nine countries across the Global North and South reveal diverging preferences on whether a benefit-sharing system should rely on monetary or non-monetary contributions, and whether governments should be responsible for payments. However, consensus emerges in favor of criteria-based DSI allocations and designated funding purposes.

The Convention on Biological Diversity (CBD) and its Nagoya Protocol established guidelines to ensure that benefits from the use of genetic resources are shared in a fair and equitable manner^1–4^. However, designing an access and benefit-sharing (ABS) system that is politically feasible, socially acceptable, and economically viable remains a challenge^2,5–9^. While the political process of shaping ABS rules has been relatively slow, technological advancements are progressing rapidly, which risks creating a disconnect between emerging technologies and existing regulations. A notable challenge is that essential genetic information is now accessible in digital formats, fundamentally altering the dynamics of accessing biodiversity from a regulatory perspective. In 2022, during the 15^th^ Conference of Parties (COP15) to the CBD, an Ad Hoc Open-Ended Working Group on Benefit-Sharing from the Use of Digital Sequence Information (DSI) on Genetic Resources was established. This group aimed to develop a multilateral benefit-sharing mechanism related to the use of digital sequences at COP16 in 2024^2,5,10^.

DSI accounts for digital representations of genetic material from animals, plants, and microorganisms, used in diverse fields such as medical research, drug development, and food production^11^. Industries like pharmaceuticals, cosmetics, plant and animal breeding, or biotechnology, which heavily rely on DSI, generate annual revenues ranging from one to a few trillion USD. Even a small share of this revenue could provide significant financial benefits to the countries and communities where these genetic resources originate^12^. In particular, the Global South, along with indigenous peoples and local communities, often lack access to infrastructure and technologies to fully access, analyze, generate, utilize, and store DSI. Consequently, most of the benefits derived from DSI tend to remain concentrated in the Global North^13,14^.

In light of these disparities, COP16, which took place in Colombia in late 2024, addressed critical operational questions related to DSI, such as who will bear the costs, the amount of revenue-sharing, the conditions for payments, and how to ensure transparency and inclusivity in the decision-making process^10,15^. A central concern surrounding DSI governance is how perspectives from civil society, and indigenous groups are not reflected in the proposals for negotiation^16,17^. The rather technical nature of DSI may explain why the topic receives little attention outside a small group of experts. However, if this process remains exclusive and disconnected from broader societal input, there is a risk that any attempts to formalize a multilateral DSI benefit-sharing mechanism could face sudden political backlash and insufficient public support. This raises important questions: if civil society were to be surveyed for their views on multilateral benefit-sharing, what preferences would emerge? Would these vary across countries, across continents, and between Global North and South?.

To address these questions, we conducted a multi-country survey in March 2024 on individual preferences for the different elements of a multilateral benefit-sharing mechanism for DSI (Fig. 1a for list of countries). We had 2,619 participants in total (Supplementary Tables 1–3 for the actual number of participants per country). In the survey, we present individuals with a pair of competing alternatives for a benefit-sharing mechanism for DSI and ask them to distribute a total of 100 points between the two alternatives (Fig. 1b for the alternatives and the supplementary material for the survey in English). The more points an individual distributes to an alternative, the more they agree with this alternative. The total distributed points are required to sum up to 100. More information is provided in the Methods section.

**Fig. 1:**
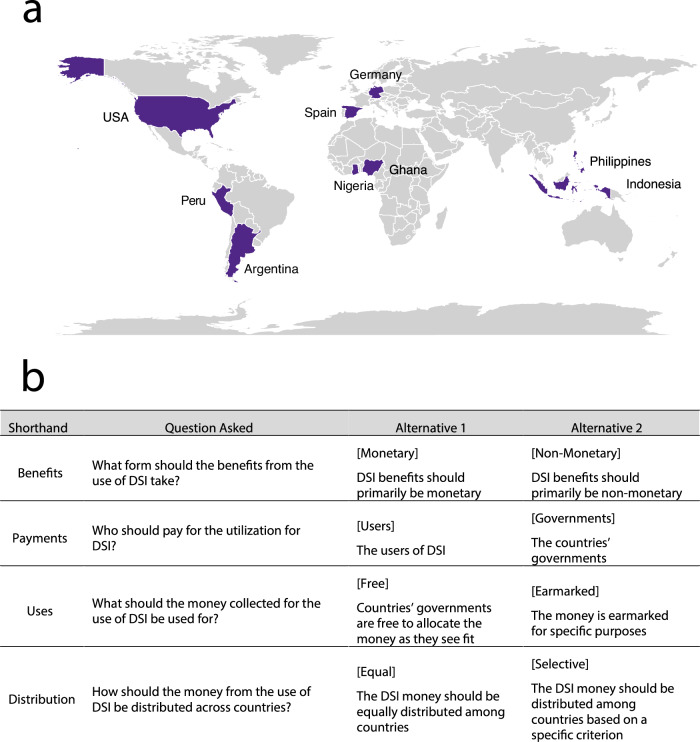
Assessing citizens’ preferences on the design of a multilateral benefit-sharing mechanism for digital sequence information on genetic resources. **a** Individuals in nine countries were surveyed regarding their preferences for the different elements of a multilateral mechanism for sharing benefits from the use of DSI. **b** Survey questions and the pair of competing alternatives to which survey respondents can distribute 100 points. The words in square brackets are shorthand names for each alternative.

The survey questions highlight key policy debates surrounding the structure of a multilateral benefit-sharing mechanism for DSI^2,7,9^. First, respondents are asked whether benefits from the use of DSI should be primarily monetary or non-monetary, such as through capacity building or research partnerships (“Benefits” in Fig. 1b). Next, respondents are asked who should fund the use of DSI – whether it should be the users of DSI or governments, with the latter retaining the option to recoup payments from domestic industries (“Payments” in Fig. 1b)^7^. Another question addresses whether receiving governments should have unrestricted use of potential funds coming from the utilization of DSI or if the funds should be earmarked for specific purposes, like biodiversity conservation (“Uses” in Fig. 1b). Finally, participants are asked whether monetary payments should be equally distributed across countries or allocated based on certain criteria, such as a country’s biodiversity richness or conservation efforts (“Distribution” in Fig. 1b). While respondents could express varying degrees of support for either side, our survey outcomes can be interpreted in terms of majority preference.

Across nine countries spanning five continents, we observe diverse preferences both within and across countries, continents, and between Global North and South (Fig. 2, Supplementary Figs. 1–9, and Supplementary Tables 1–3). Despite this variation, some results emerge consistently across geographic locations. First, we find that, regardless of country, the median respondent tends to be indecisive between the alternatives, often splitting their allocation evenly at 50-50, irrespective of the survey question (Fig. 2b). Additionally, there is broad consensus on the distribution of funds from DSI: respondents generally favor allocating funds to countries based on specific criteria and earmarking them for designated purposes within each country. This is evident in Fig. 2a, where average responses for “Free” and “Equal” under the questions for “Uses” and “Distribution”, respectively, are lower than median, and further illustrated by the left-skewed distributions for “Uses” and “Distributions” in Fig. 2b. This result is also supported by Supplementary Tables 7, 8. After excluding respondents who were indecisive, we not only find more individuals on left side of the distribution (Supplementary Table 7) but also find them distributing more points towards the left side of the distribution (Supplementary Table 8).

**Fig. 2:**
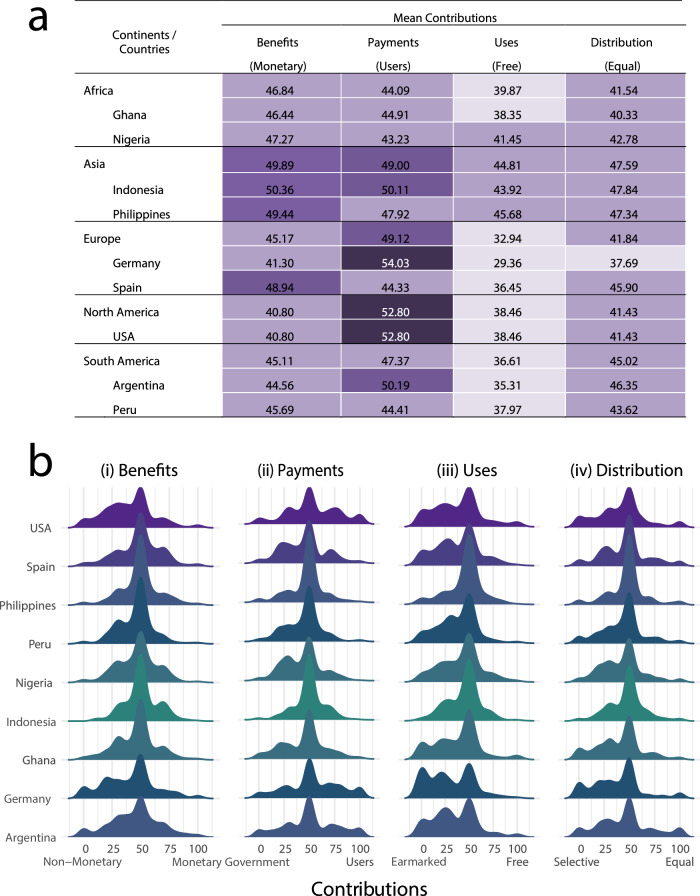
Policy preferences of citizens across countries. **a** Average support for each of the elements of a multilateral benefit-sharing mechanism for DSI. Under each element, the words in parenthesis are the alternatives that the averages pertain to. Averages below 40 are considered very low and are shaded in pale purple, averages between 41 and 48 are considered low and are shaded in light purple, averages between 48 and 52 are considered median values and are shaded in medium purple, and averages above 52 are considered high values and are shaded in dark purple. **b** Distributions of contributions across countries for the alternative i) “DSI benefits should primarily be monetary”, ii) “The users of DSI”, iii) “Countries’ governments are free to allocate the money as they see fit”, iv) “The DSI money should be equally distributed among countries”. All differences across countries for alternatives are statistically significantly different from one another (Kruskal–Wallis Test, *p*-value = 0.0001).

As for the questions on whether benefits from the use of DSI should take a monetary or non-monetary form and on whether the users of DSI or each countries’ governments should pay for the utilization of DSI, we find no consensus. Germany and the US stand out as the countries with a strongest preference to have non-monetary benefits while Indonesia is the only country where the majority favors monetary payments (Fig. 2, Supplementary Tables 6, 8). On average, respondents in the Global South are more supportive towards monetary benefits (Supplementary Table 4, 5). As for who should pay for the utilization of DSI, in Germany, the US, Argentina, and Indonesia the majority favors the users of DSI to pay for the utilization of DSI while Nigeria, Ghana, Spain, Peru, and the Philippines are in favor of governments paying (Fig. 2). On average, respondents in the Global South are more supportive towards governments paying for DSI (Supplementary Tables 4, 5). We also looked at how polarized preferences are within countries (Supplementary Tables 7, 8) and find preferences to be polarized in Germany and the USA, where more citizens indicated preferences to the very left or right of the spectrum (Supplementary Table 8).

If civil society were surveyed on multilateral benefit-sharing for DSI on genetic resources, preferences would vary across countries, continents, and between the Global North and South. While responses showed significant diversity, some clear patterns emerged. There was no global consensus on whether benefits should be primarily monetary or non-monetary, such as capacity building or research partnerships. However, even if Indonesia was the only country where the majority favored monetary payments, the Global South generally showed a stronger preference for monetary benefits, while Germany and the U.S. leaned more toward non-monetary forms.

Similarly, opinions differed on who should bear the costs of utilizing DSI. On average, the Global South leaned toward government funding, while the Global North supported having users cover the costs. Particularly, respondents in Germany, the U.S., Argentina, and Indonesia preferred that DSI users pay, whereas Nigeria, Ghana, Spain, Peru, and the Philippines favored government-funded mechanisms. Despite these differences, there was broad agreement that funds derived from DSI should be earmarked for specific purposes, such as biodiversity conservation, rather than being freely allocated by governments. Likewise, respondents preferred the distribution of funds to be based on specific criteria—such as a country’s biodiversity richness—rather than being distributed among all countries in equal share.

These preferences also varied across geographic regions. While respondents from Africa, Asia, and South America leaned toward monetary benefits, the ones from Europe and North America showed a greater preference for non-monetary alternatives. Country-specific variations were also evident, with Germany and the U.S. displaying more polarized views, as respondents in these nations tended to have stronger preferences at either end of the spectrum.

These results highlight the complexity of achieving consensus in international agreements and emphasize the importance of designing an inclusive, responsive framework for benefit-sharing from the use of DSI that acknowledges both global and regional differences. They underscore the need for transparent and participatory discussions that specifically include civil society perspectives, ensuring final agreements that reflect the diversity of global preferences while supporting the equitable distribution of benefits arising from the use of DSI. As discussions continue, our findings can guide policymakers toward a more inclusive and effective multilateral agreement that represents civil society interests, which are often underrepresented in these negotiations. Such an approach may help bridge regional disparities in DSI access and benefit distribution, fostering a sustainable model that honors the contributions and priorities of countries, communities, and civil society worldwide.

## Methods

We use survey data collected in 9 different countries for our analyses. During sampling, the only criterion for inclusion was respondents to be citizens and residents of the country where they took the survey. Country selection was based on the following factors: (1) the availability of a survey panel with at least 300 participants per country, (2) the distribution of countries across multiple continents, (3) varying levels of biodiversity richness, (4) representation from both the Global North and the Global South, (5) access to translators for survey translation into the local language, and (6) countries with a coastline (as the survey was embedded in a bigger survey on marine biodiversity). As a result, some countries with richer biodiversity than our sample of 9 countries have not been included in our survey.

The data collection was conducted between February 26, 2024 and March 01, 2024 using the consumer panel (i.e., participant database) of Norstat Netherlands. We provided Norstat Netherlands with a survey link, which they then distributed to individuals within their panel. The only requirement we specified was that the link be sent to participants aged 18 and older. We assume that our survey participants are non-experts in the subject of the survey, given that they were recruited from the consumer panel of Norstat Netherlands.

The survey was originally written in English and programmed in Qualtrics. It was translated to Spanish, German, Filipino, and Indonesian using the translation function in Qualtrics. These translations were then reviewed and revised by local speakers of Spanish, German, Filipino, and Indonesia. The translated surveys were re-translated back to English to check each translation’s comparability with the original English version. The English version of the survey was submitted for approval to the Wageningen University & Research Human Subjects Board (IRB Approval Number 2023-031) and is available in the Supplementary Material. A preregistration of the project was submitted in February 2024 (AsPredicted #163538).

## Supplementary information

Supplementary Materials

## Data Availability

Raw data in CSV format along with processed data in DTA format is available via Open Science Framework, https://osf.io/hdp65/.

## Code availability

The R Scripts and the Stata do-files, codes needed to reproduce all the results of the main manuscript and the supplementary material is available via Open Science Framework, https://osf.io/hdp65/.
