# Supplementary material for: Citizen consensus and diverging views on benefit-sharing for genetic resources
Source: NPJ Biodivers. 2025 Jul 3;4:26. doi: 10.1038/s44185-025-00093-7 (PMC12226334; doi:10.1038/s44185-025-00093-7)
Supplement: Supplementary file 1 — Supplementary Materials [file 44185_2025_93_MOESM1_ESM.pdf]

Supplementary Materials for

Citizen Consensus and Diverging Views on Benefit-Sharing for Genetic Resources

Anna Lou Abatayo, Xiaolongren Ding, Esteban Neira-Monsalve, Andries Richter

Correspondence to: anna.abatayo@wur.nl

This PDF file includes:

Supplementary Fig. 1 to 9
Supplementary Tab. 1 to 8
Survey Questionnaire

Supplementary Fig. 1.
Significant Pairwise Differences Across Contributions.

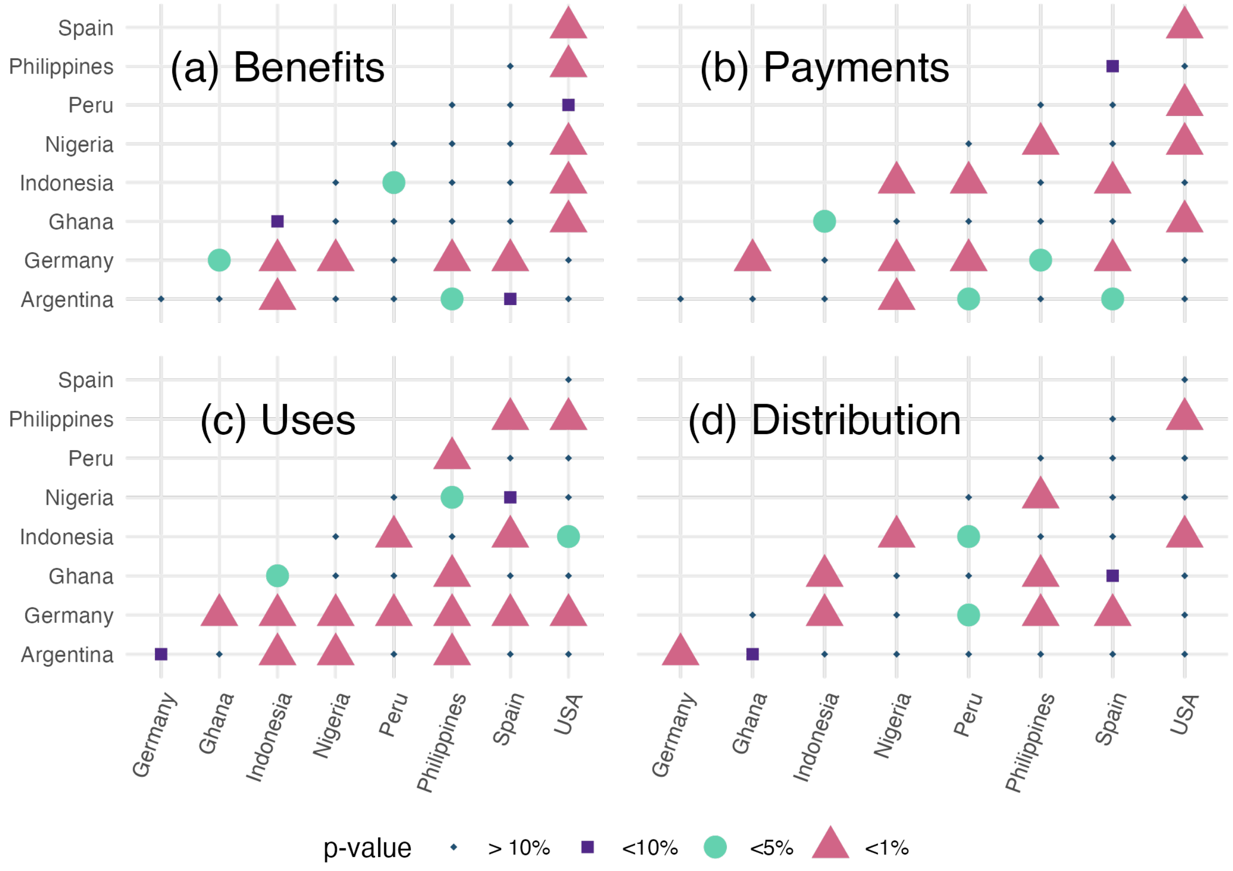

p-values for post hoc Dunn tests with Bonferroni correction. Similar graphs for the *p*-values of two-tailed t-tests can be found in Fig. S3 of the supplementary material. **a,** *p*-value for pairwise comparisons between countries for the alternative "DSI benefits should primarily be monetary". **b,** *p*-value for pairwise comparisons between countries for the alternative "The users of DSI". **c,** *p*-value for pairwise comparisons between countries for the alternative "Countries' governments are free to allocate the money as they see fit". **d,** *p*-value for pairwise comparisons between countries for the alternative "The DSI money should be equally distributed among countries".

Supplementary Fig. 2.

Countries Differ in Their Distribution of Points.

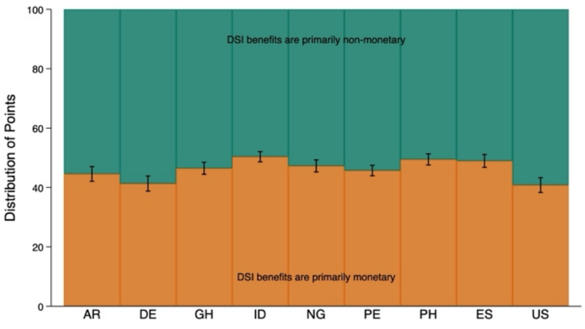

(a) Benefits

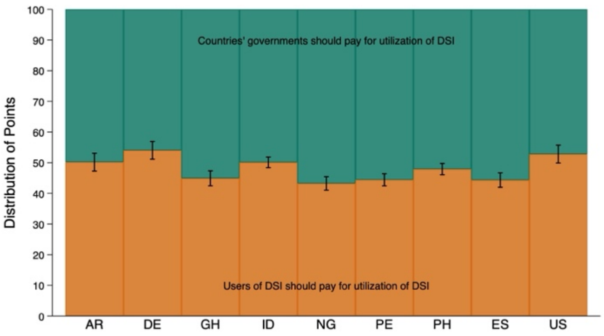

(b) Payments

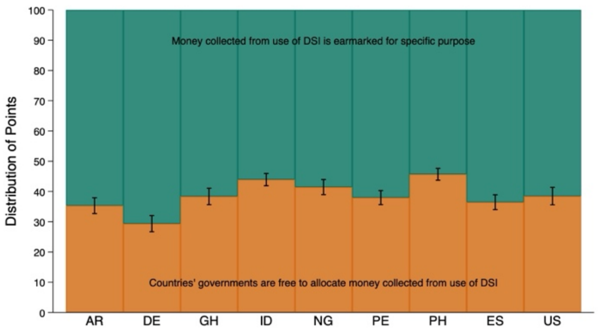

(c) Uses

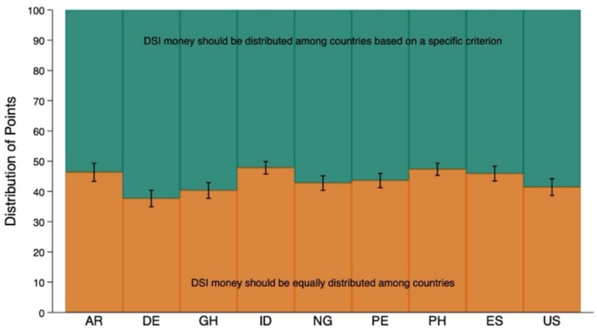

(d) Distribution

Bars represent the average distribution 100 points between alternatives across countries, along with 95% confidence intervals (whiskers). The bottom (orange) bar is the average distribution for Alternative 1 while the top (blue) bar is the average allocation for Alternative 2. Fig. 1b in the main manuscript lists what these alternatives are. **a,** Question on “What form should the benefits from the use of DSI take?”. **b,** Question on “Who should pay for the utilization for DSI?”. **c,** Question on “What should the money collected for the use of DSI be used for?”. **d,** Question on “How should the money from the use of DSI be distributed across countries?”.

Supplementary Fig. 3.

t-Tests: Countries Differ in Their Distribution of Points.

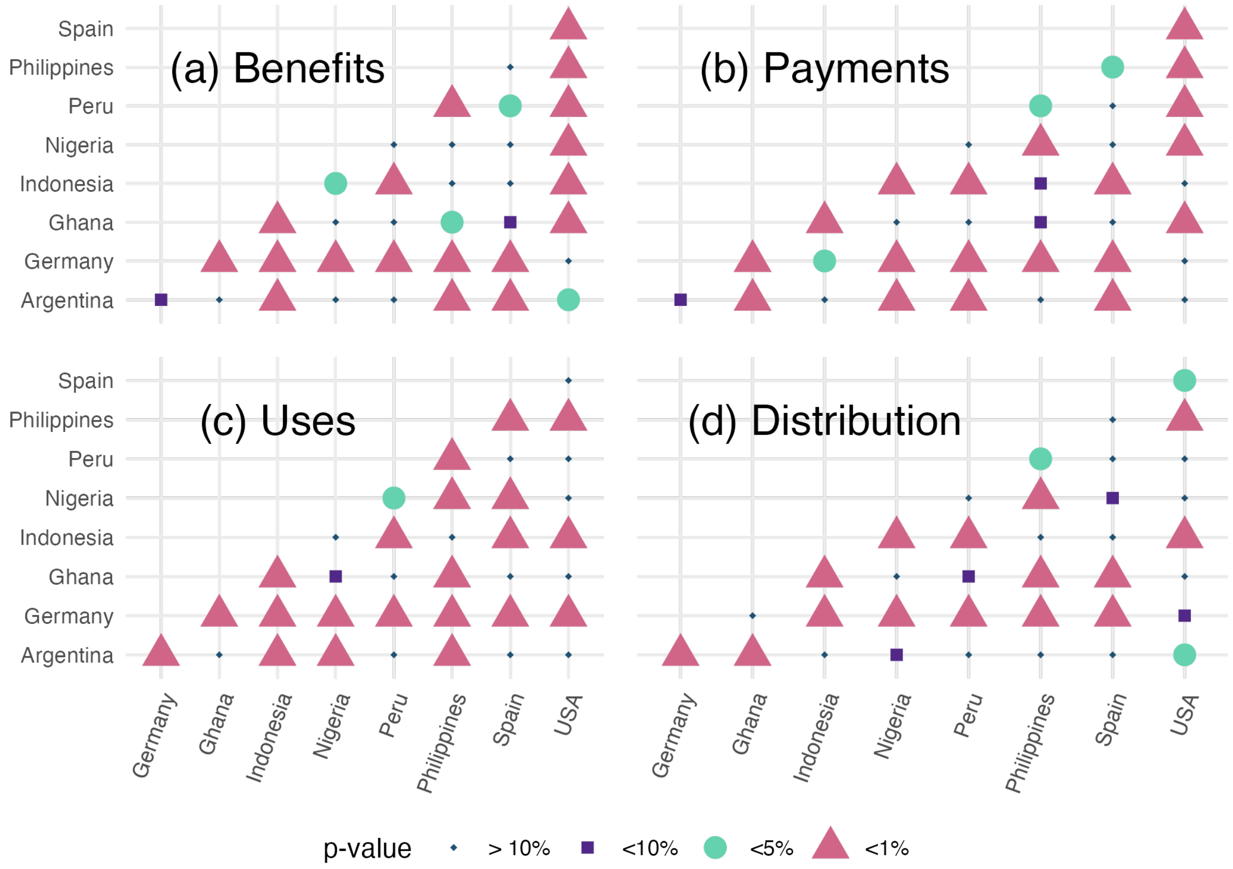

p-values from a two-sided Student's t-Test. Similar graphs for the *p*-values for post hoc Dunn tests with Bonferroni correction Fig. S1 of the supplementary material. **a,** *p*-value for pairwise comparisons between countries for the alternative "DSI benefits should primarily be monetary". **b,** *p*-value for pairwise comparisons between countries for the alternative "The users of DSI". **c,** *p*-value for pairwise comparisons between countries for the alternative "Countries' governments are free to allocate the money as they see fit". **d,** *p*-value for pairwise comparisons between countries for the alternative "The DSI money should be equally distributed among countries".

Supplementary Fig. 4.

Heterogeneous Distributions of Contributions, by Hemisphere.

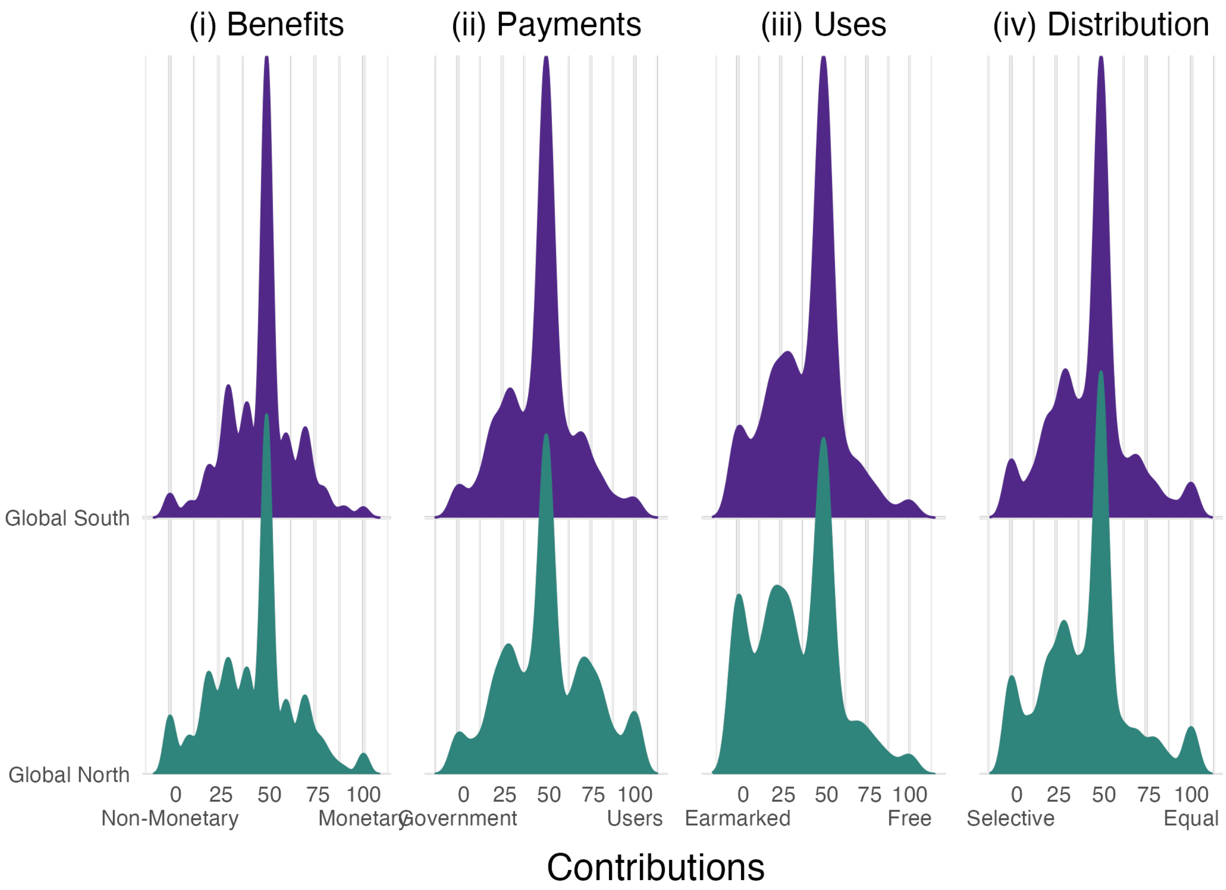

Argentina, Ghana, Indonesia, Nigeria, Peru, and the Philippines are considered Global South countries while are Germany, Spain, and the US are considered Global North countries. **a,** Distributions of contributions between hemispheres for the survey question "What form should the benefits from the use of DSI take?" (Kruskal-Wallis Test, *p*-value = 0.0001). **b,** Distributions of contributions between hemispheres for the survey question "Who should pay for the utilization of DSI?" (Kruskal-Wallis Test, *p*-value = 0.0003). **c,** Distributions of contributions between hemispheres for the survey question "What should the money collected for the use of DSI be used for?" (Kruskal-Wallis Test, *p*-value = 0.0001). **d,** Distributions of contributions between hemispheres for the survey question "How should the money from the use of DSI be distributed across countries?" (Kruskal-Wallis Test, *p*-value = 0.0001). Fig. S2 in the supplementary material shows a stacked bar graph of the average distribution of points per country per question asked.

Supplementary Fig. 5.

Global South and North Countries Differ in Their Distribution of Points.

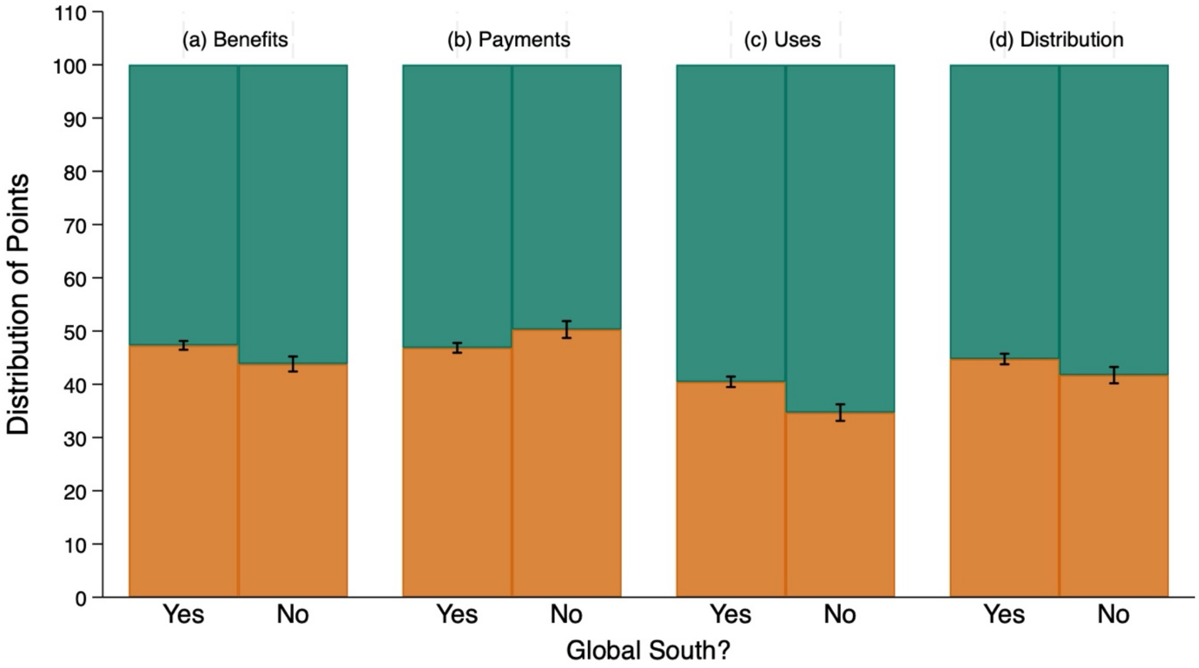

Argentina, Ghana, Indonesia, Nigeria, Peru, and the Philippines are considered Global South countries while are Germany, Spain, and the US are considered Global North countries. Bars represent the average distribution 100 points between alternatives across countries, along with 95% confidence intervals (whiskers). The bottom (orange) bar is the average distribution for Alternative 1 while the top (blue) bar is the average allocation for Alternative 2. Fig. 1b in the main manuscript lists what these alternatives are. Student's t-test show differences across contributions between individuals in Global South and North countries for the questions: **a,** "What form should the benefits from the use of DSI take?" (*p*-value = 0.0000). **b,** "Who should pay for the utilization for DSI?" (*p* -value = 0.0001). **c,** "What should the money collected for the use of DSI be used for?" (*p* -value = 0.0000). **d,** "How should the money from the use of DSI be distributed across countries?" (*p*-value = 0.0009).

Supplementary Fig. 6.

Heterogeneous Distributions of Contributions Across Continents.

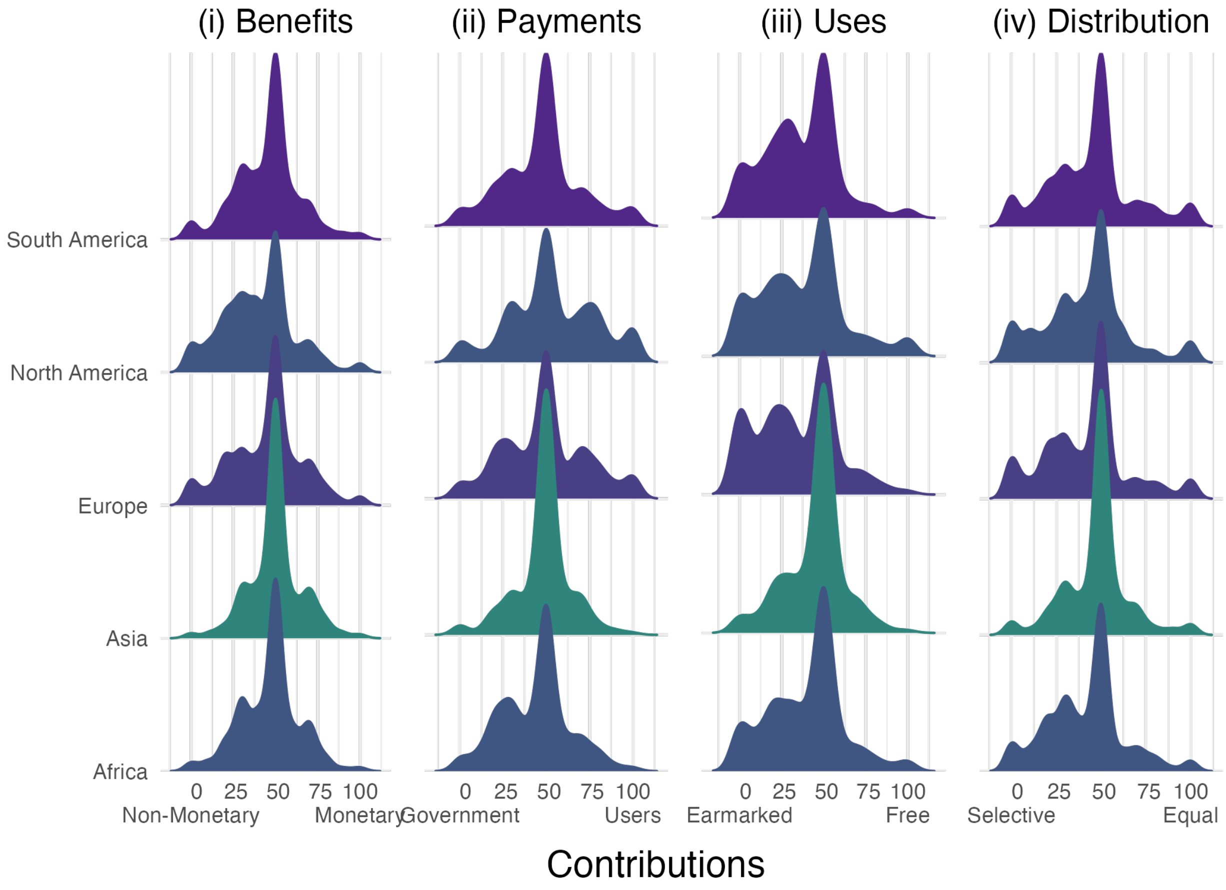

a, Distributions of contributions between hemispheres for the survey question “What form should the benefits from the use of DSI take?” (Kruskal-Wallis Test, *p*-value = 0.0001). **b,** Distributions of contributions between hemispheres for the survey question “Who should pay for the utilization of DSI?” (Kruskal-Wallis Test, *p*-value = 0.0001). **c,** Distributions of contributions between hemispheres for the survey question “What should the money collected for the use of DSI be used for?” (Kruskal-Wallis Test, *p*-value = 0.0001). **d,** Distributions of contributions between hemispheres for the survey question “How should the money from the use of DSI be distributed across countries?” (Kruskal-Wallis Test, *p*-value = 0.0001).

Supplementary Fig. 7.

Continents Differ in Their Distribution of Points.

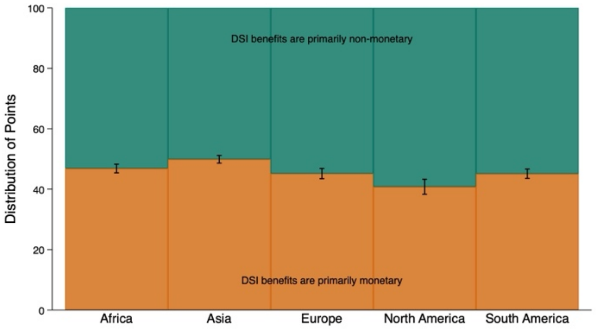

(a) Benefits

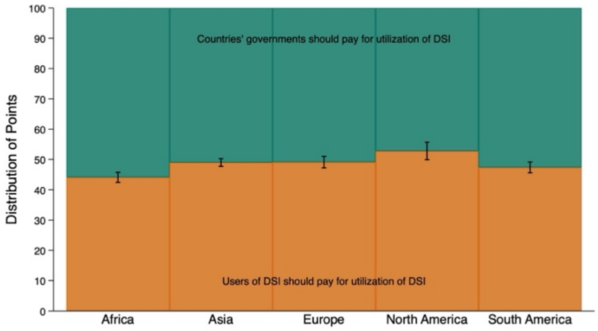

(b) Payments

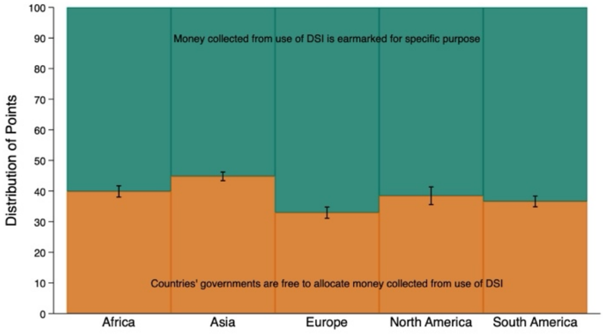

(c) Uses

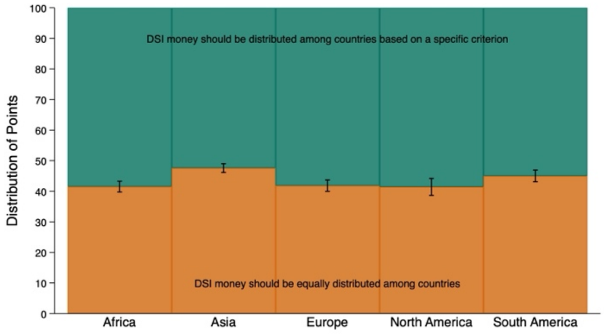

(d) Distribution

Africa is composed of Ghana and Nigeria, Asia is composed of Indonesia and the Philippines, Europe is composed of Germany and Spain, North America is composed of the US, and South America is composed of Argentina and Peru. Bars represent the average distribution 100 points between alternatives across countries, along with 95% confidence intervals (whiskers). The bottom (orange) bar is the average distribution for Alternative 1 while the top (blue) bar is the average allocation for Alternative 2. Tab. 1 in the main manuscript lists what these alternatives are. **a,** Question on “What form should the benefits from the use of DSI take?”. **b,** Question on “Who should pay for the utilization for DSI?”. **c,** Question on “What should the money collected for the use of DSI be used for?”. **d,** Question on “How should the money from the use of DSI be distributed across countries?”.

Supplementary Fig. 8.

Significant Pairwise Differences Across Contributions.

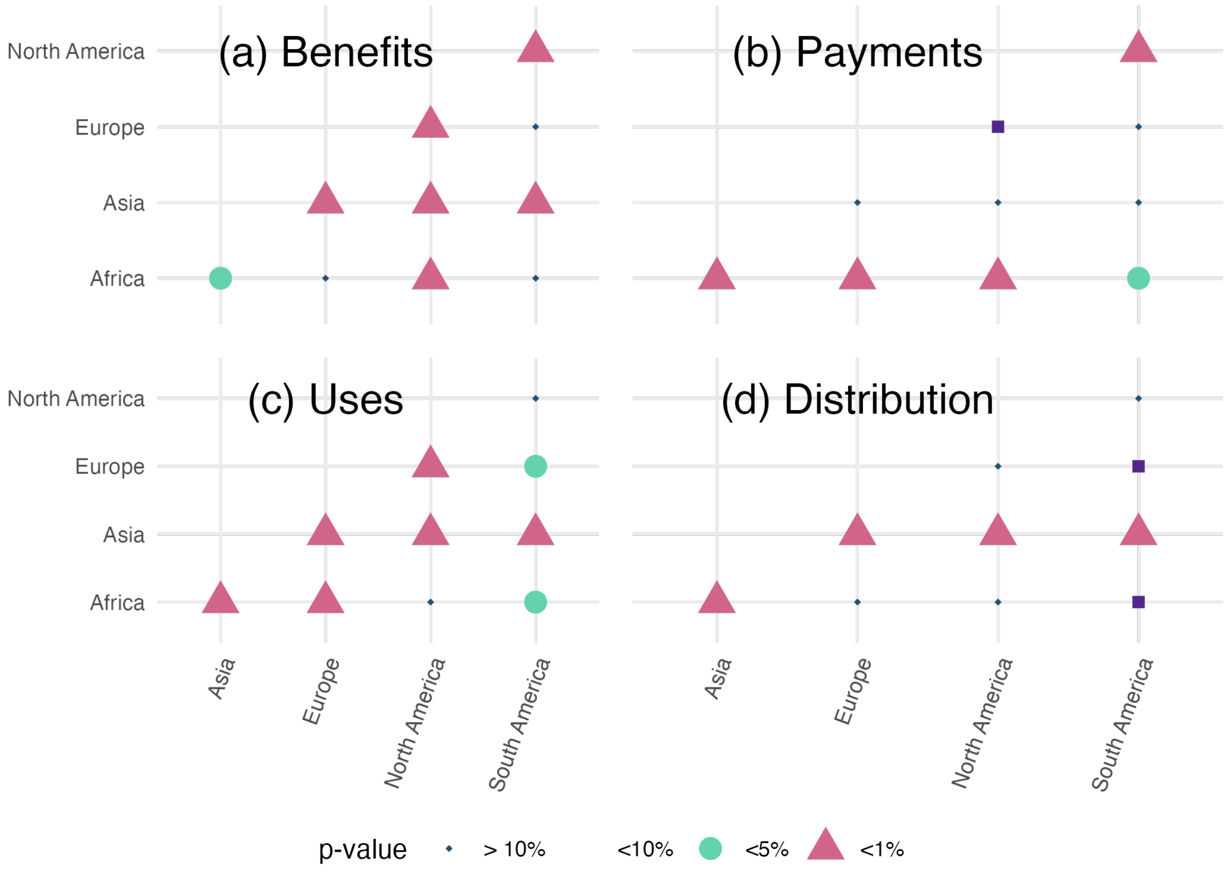

Africa is composed of Ghana and Nigeria, Asia is composed of Indonesia and the Philippines, Europe is composed of Germany and Spain, North America is composed of the US, and South America is composed of Argentina and Peru. *p*-values for post hoc Dunn tests with Bonferroni correction. Similar graphs for the *p*-values of two-tailed t-tests can be found in Figs. S9 in the Supplementary Information. **a,** *p*-value for pairwise comparisons between countries for the alternative “DSI benefits should primarily be monetary”. **b,** *p*-value for pairwise comparisons between countries for the alternative “The users of DSI”. **c,** *p*-value for pairwise comparisons between countries for the alternative “Countries’ governments are free to allocate the money as they see fit”. **d,** *p*-value for pairwise comparisons between countries for the alternative “The DSI money should be equally distributed among countries”.

Supplementary Fig. 9.

t-Tests: Continents Differ in Their Distribution of Points.

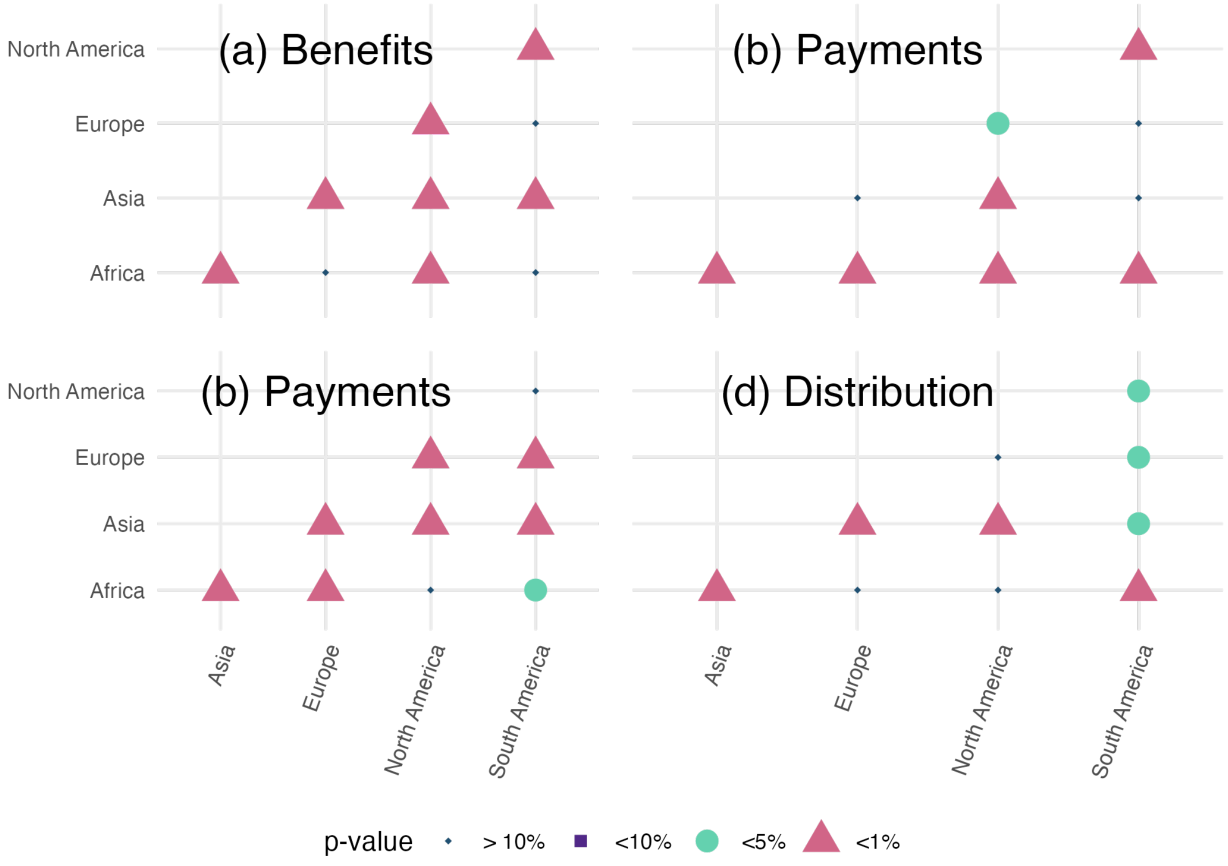

Africa is composed of Ghana and Nigeria, Asia is composed of Indonesia and the Philippines, Europe is composed of Germany and Spain, North America is composed of the US, and South America is composed of Argentina and Peru. *p*-values from a two-sided Student's t-Test. Similar graphs for the *p*-values for post hoc Dunn tests with Bonferroni correction Fig. S8 of the supplementary material. **a,** *p*-value for pairwise comparisons between countries for the alternative "DSI benefits should primarily be monetary". **b,** *p*-value for pairwise comparisons between countries for the alternative "The users of DSI". **c,** *p*-value for pairwise comparisons between countries for the alternative "Countries' governments are free to allocate the money as they see fit". **d,** *p*-value for pairwise comparisons between countries for the alternative "The DSI money should be equally distributed among countries".

Supplementary Tab. 1.
Number of Observations, by Hemisphere

Category	Observations		Mean Contributions			
	Recruited	Final	Benefits	Payments	Uses	Distribution
Global South	1,906	1,746	47.29	46.83	40.45	44.74
Global North	988	873	43.8	50.27	34.67	41.71
**Category**	**Statistics**		**Benefits**	**Payments**	**Uses**	**Distribution**
Global South	Mean Contributions		45.49	44.51	34.25	41.18
	Observations		(1,049)	(1,007)	(1,059)	(1,042)
Global North	Mean Contributions		40.74	50.41	28.56	37.17
	Observations		(585)	(591)	(624)	(564)

Notes: Observations were dropped if individuals failed to finish answering the entire survey within a week and if Qualtrics detected an observation is either duplicated or fraudulent. Ghana, Nigeria, Indonesia, the Philippines, Argentina, and Peru are considered Global South countries. Germany, Spain, and the USA are considered Global North countries. “Benefits”, “Payments”, “Uses”, and “Distribution” are average individual distribution of points to Alternative 1. Fig. 1b in the main manuscript lists what these alternatives are. The top part of the table are averages under the different DSI elements while the bottom part of the table are the same averages after excluding individuals who were indecisive between the two alternatives (i.e., contributed 50).

Supplementary Tab. 2.

Number of Observations, by Continent.

Continent	Observations		Mean Contributions			
	Recruited	Final	Benefits	Payments	Uses	Distribution
Africa	661	576	46.84	44.09	39.87	41.54
Asia	624	588	49.89	49	44.81	47.59
Europe	653	599	45.17	49.12	32.94	41.84
North America	335	274	40.8	52.8	38.46	41.43
South America	621	582	45.11	47.37	36.61	45.02
**Continent**	**Statistics**		**Benefits**	**Payments**	**Uses**	**Distribution**
Africa	Mean Contributions		45.01	40.87	34.23	37.14
	Observations		(364)	(373)	(370)	(379)
Asia	Mean Contributions		49.79	47.83	39.73	45.09
	Observations		(308)	(271)	(297)	(289)
Europe	Mean Contributions		42.77	48.69	26.18	37.04)
	Observations		(400)	(402)	(429)	(377)
North America	Mean Contributions		36.37	54.06	33.78	37.44
	Observations		(185)	(189)	(195)	(187)
South America	Mean Contributions		42.46	45.78	30.11	42.25
	Observations		(377)	(363)	(392)	(374)

Notes: Observations were dropped if individuals failed to finish answering the entire survey within a week and if Qualtrics detected an observation is either duplicated or fraudulent. Africa is composed of Ghana and Nigeria, Asia is composed of Indonesia and the Philippines, Europe is composed of Germany and Spain, North America is composed of the US, and South America is composed of Argentina and Peru. “Benefits”, “Payments”, “Uses”, and “Distribution” are average individual distribution of points to Alternative 1. Fig. 1b in the main manuscript lists what these alternatives are. The top part of the table are averages under the different DSI elements while the bottom part of the table are the same averages after excluding individuals who were indecisive between the two alternatives (i.e., contributed 50).

Supplementary Tab. 3.

Number of Observations, by Country.

Country	Code	Observations		Mean Contributions			
		Recruited	Final	Benefits	Payments	Uses	Distribution
Argentina	AR	308	298	44.56	50.19	35.31	46.35
Germany	DE	335	296	41.30	54.03	29.36	37.69
Ghana	GH	341	293	46.44	44.91	38.35	40.33
Indonesia	ID	309	290	50.36	50.11	43.92	47.84
Nigeria	NG	320	283	47.27	43.23	41.45	42.78
Peru	PE	313	284	45.69	44.41	37.97	43.62
Philippines	PH	315	298	49.44	47.92	45.68	47.34
Spain	ES	318	303	48.94	44.33	36.45	45.90
USA	US	335	274	40.80	52.80	38.46	41.43
**Country**	**Code**	**Statistics**		**Benefits**	**Payments**	**Uses**	**Distribution**
Argentina	AR	Mean Contributions		42.67	50.28	29.44	44.67
		Observations		(221)	(201)	(213)	(204)
Germany	DE	Mean Contributions		37.8	55.81	22.47	31.96
		Observations		(211)	(205)	(222)	(202)
Ghana	GH	Mean Contributions		43.97	41.81	31.14	35.17
		Observations		(173)	(182)	(181)	(191)
Indonesia	ID	Mean Contributions		50.65	50.22	39.12	46.18
		Observations		(159)	(147)	(162)	(164)
Nigeria	NG	Mean Contributions		45.95	39.97	37.19	39.14
		Observations		(191)	(191)	(189)	(191)
Peru	PE	Mean Contributions		42.16	40.2	30.92	39.35
		Observations		(156)	(162)	(179)	(170)
Philippines	PH	Mean Contributions		48.87	44.99	40.46	43.66
		Observations		(149)	(124)	(135)	(125)
Spain	ES	Mean Contributions		48.31	41.27	30.16	42.91
		Observations		(189)	(197)	(207)	(175)
USA	US	Mean Contributions		36.37	54.06	33.78	37.44
		Observations		(185)	(189)	(195)	(187)

Notes: Observations were dropped if individuals failed to finish answering the entire survey within a week and if Qualtrics detected an observation is either duplicated or fraudulent. "Benefits", "Payments", "Uses", and "Distribution" are average individual distribution of points to Alternative 1. Fig. 1b in the main manuscript lists what these alternatives are. The top part of the table are averages under the different DSI elements while the bottom part of the table are the same averages after excluding individuals who were indecisive between the two alternatives (i.e., contributed 50).

Supplementary Tab. 4.

Regression Results: Global North vs. Global South.

Dep. Var.:	Benefits		Payments		Uses		Distribution	
	(1)	(2)	(3)	(4)	(5)	(6)	(7)	(8)
Global South	3.4960***	2.3572**	-3.4404***	-1.9963	5.7726***	2.5986*	3.0223***	0.4863
	(0.8279)	(1.1556)	(0.9330)	(1.3332)	(0.9351)	(1.3569)	(0.9338)	(1.4176)
Constant	43.7973***	43.6239***	50.2749***	59.7708***	34.6735***	50.2496***	41.7136***	42.3935***
	(0.7144)	(4.1389)	(0.8085)	(4.8705)	(0.7908)	(4.6860)	(0.7844)	(4.2941)
Controls	No	Yes	No	Yes	No	Yes	No	Yes
R-squared	0.01	0.05	0.01	0.05	0.02	0.07	0.00	0.03
Obs.	2,619	2,285	2,619	2,285	2,619	2,285	2,619	2,285

Notes: Ordinary least squares regression with robust standard errors. Baseline category are observations from Global North countries. “Benefits”, “Payments”, “Uses”, and “Distribution” are individual contributions to Alternative 1. Fig. 1b in the main manuscript lists what these alternatives are. Controls include gender, number of children, educational level, confidence levels (on government, big corporations, environmental organizations, and new technologies), preference between protecting the environment and economic growth, and beliefs on how exaggerated environmental threats are. * $p<0.10$; ** $p<0.05$; *** $p<0.01$.

Supplementary Tab. 5.

Regression Results, by Continent.

Dep. Var.:	Benefits		Payments		Uses		Distribution	
	(1)	(2)	(3)	(4)	(5)	(6)	(7)	(8)
Asia	3.0474***	2.3593*	4.9115***	3.8264***	4.9414***	3.8397**	6.0502***	5.4182***
	(0.9734)	(1.2648)	(1.0576)	(1.3696)	(1.1825)	(1.5297)	(1.1559)	(1.4943)
Europe	-1.6751	-0.5491	5.0334***	3.5601**	-6.9266***	-4.5929**	0.3065	2.8449
	(1.1263)	(1.5571)	(1.2768)	(1.7569)	(1.3218)	(1.8599)	(1.3049)	(1.8563)
N. America	-6.0445***	-4.9688***	8.7125***	6.6157***	-1.4136	-0.4601	-0.1112	3.2622
	(1.4608)	(1.8631)	(1.6919)	(2.1542)	(1.7438)	(2.2232)	(1.6681)	(2.1665)
S. America	-1.7303	-0.2492	3.2809***	3.1579**	-3.2633**	-2.7185*	3.4807***	4.1956***
	(1.0680)	(1.3149)	(1.2322)	(1.5298)	(1.2957)	(1.5640)	(1.3266)	(1.5912)
Constant	46.8437***	45.8691***	44.0868***	54.5032***	39.8698***	50.9729***	41.5382***	39.1289***
	(0.7274)	(4.0036)	(0.8365)	(4.8009)	(0.9404)	(4.6606)	(0.8987)	(4.3336)
Controls	No	Yes	No	Yes	No	Yes	No	Yes
R-squared	0.02	0.06	0.01	0.05	0.04	0.08	0.01	0.04
Obs.	2,619	2,285	2,619	2,285	2,619	2,285	2,619	2,285

Notes: Ordinary least squares regression with robust standard errors. Baseline category are observations from individuals in “Africa”. “Benefits”, “Payments”, “Uses”, and “Distribution” are individual contributions to Alternative 1. Fig. 1b in the main manuscript lists what these alternatives are. Controls include gender, number of children, educational level, confidence levels (on government, big corporations, environmental organizations, and new technologies), preference between protecting the environment and economic growth, and beliefs on how exaggerated environmental threats are. * $p<0.10$; ** $p<0.05$; *** $p<0.01$.

Supplementary Tab. 6.

Regression Results, by Country.

Dep. Var.:	Benefits		Payments		Uses		Distribution	
	(1)	(2)	(3)	(4)	(5)	(6)	(7)	(8)
GE	-3.2563*	-3.7427*	3.8391*	2.2534	-5.9506***	-3.7638	-8.6598***	-6.2964**
	(1.8124)	(2.1949)	(2.0795)	(2.6169)	(1.8933)	(2.3859)	(2.0621)	(2.6169)
GH	1.8765	0.2702	-5.2767***	-5.7779***	3.0428	4.0141*	-6.0145***	-5.9741***
	(1.6319)	(1.8763)	(1.9257)	(2.2237)	(1.9131)	(2.1362)	(2.0127)	(2.2387)
ID	5.7982***	3.4929*	-0.0776	-1.2256	8.6153***	6.1207***	1.4924	-0.0985
	(1.5430)	(1.8176)	(1.7181)	(2.0311)	(1.6796)	(2.0014)	(1.8454)	(2.1688)
NG	2.7046*	1.9094	-6.9547***	-8.0036***	6.1399***	6.5777***	-3.5645*	-2.1708
	(1.6350)	(2.0931)	(1.8475)	(2.3888)	(1.8337)	(2.4471)	(1.9516)	(2.4860)
PE	1.1333	0.9113	-5.7795***	-6.5524***	2.6665	4.3403**	-2.7258	-1.0016
	(1.5514)	(1.7146)	(1.7788)	(2.0095)	(1.7751)	(1.9503)	(1.9382)	(2.0991)
PH	4.8758***	2.8844*	-2.2718	-3.0147	10.3725***	9.9374***	0.9933	1.3243
	(1.5838)	(1.7097)	(1.7461)	(1.9257)	(1.6557)	(1.8119)	(1.8319)	(1.9856)
ES	4.3835***	2.9752	-5.8612***	-6.7461***	1.1435	3.3989	-0.4447	1.4748
	(1.6760)	(1.8721)	(1.8965)	(2.2230)	(1.8167)	(2.1217)	(1.9678)	(2.2890)
US	-3.7611**	-4.8735**	2.6114	0.9207	3.1508	3.8792*	-4.9220**	-2.0537
	(1.7934)	(2.0628)	(2.0816)	(2.5028)	(1.9782)	(2.3225)	(2.0736)	(2.4660)
Constant	44.5604***	45.3763***	50.1879***	60.1064***	35.3054***	46.9444***	46.3490***	44.3151***
	(1.2685)	(4.2157)	(1.4721)	(4.9297)	(1.3243)	(4.8250)	(1.5238)	(4.5668)
Controls	No	Yes	No	Yes	No	Yes	No	Yes
R-squared	0.03	0.07	0.03	0.06	0.04	0.08	0.02	0.04
Obs.	2,619	2,285	2,619	2,285	2,619	2,285	2,619	2,285

Notes: Ordinary least squares regression with robust standard errors. Baseline category are observations from individuals in Argentina. “Benefits”, “Payments”, “Uses”, and “Distribution” are individual contributions to Alternative 1. Fig. 1b in the main manuscript lists what these alternatives are. Controls include gender, number of children, educational level, confidence levels (on government, big corporations, environmental organizations, and new technologies), preference between protecting the environment and economic growth, and beliefs on how exaggerated environmental threats are. * $p<0.10$; ** $p<0.05$; *** $p<0.01$.

Supplementary Tab 7.
Number of Left-Sided and Right-Sided Individuals as Percentage of Individuals Who Are Not Indecisive.

Country	Benefits		Payments		Uses		Distribution	
	Non-Monetary	Monetary	Governments	Users	Earmarked	Free	Specified	Equal
Argentina	62.44	37.56	68.66	41.29	64.79	38.97	67.65	40.69
Germany	70.14	29.86	72.2	30.73	66.67	28.38	73.27	31.19
Ghana	63.01	36.99	59.89	35.16	60.22	35.36	57.07	33.51
Indonesia	48.43	51.57	52.38	55.78	47.53	50.62	46.95	50
Nigeria	57.07	42.93	57.07	42.93	57.67	43.39	57.98	43.62
Peru	67.31	32.69	64.81	31.48	58.66	28.49	61.76	30
Philippines	52.35	47.65	62.9	57.26	57.78	52.59	62.4	56.8
Spain	51.85	48.15	49.75	46.19	47.34	43.96	56	52
USA	74.59	25.41	73.02	24.87	70.77	24.1	73.8	25.13

Supplementary Tab 8.
Skewness Indices.

Country	Benefits			Payments			Uses			Distribution		
	Non-Monetary		Monetary	Government		Users	Earmarked		Free	Specified		Equal
Argentina	15.01	>	7.68	18.04	>	11.87	19.35	>	9.83	18.61	>	12.41
Germany	18.48	>	6.28	19.64	>	8.41	22.87	>	6.08	21.13	>	8.91
Ghana	13.38	>	7.34	15.59	>	7.88	18.63	>	9.40	15.58	>	8.59
Indonesia	8.65	<	9.31	9.74	<	10.48	11.18	>	9.25	10.38	>	9.49
Nigeria	11.37	>	7.31	12.70	>	9.02	15.11	>	8.96	13.62	>	10.46
Peru	13.08	>	5.24	14.17	>	5.42	15.90	>	5.92	14.58	>	7.28
Philippines	10.72	>	9.59	15.14	>	11.28	14.65	>	9.57	15.05	>	13.16
Spain	11.34	>	9.65	11.91	>	11.28	14.00	>	9.71	13.78	<	14.69
USA	19.09	>	5.46	17.92	>	6.70	21.04	>	5.79	19.50	>	6.13

Notes: Country-level indices for the left-side of the distribution (i.e., preference towards Alternative 2) were created using the formula $\sum_i |x_i^l - 50| * (n^l/n^T)$, where x_i^l are all individuals who preferred Alternative 2 to Alternative 1 (see Fig. 1b for the alternatives), n^l is the number of individuals who preferred Alternative 2 to Alternative 1, and n^T is the total number of individuals in a country who were not indecisive between the two alternatives. Country-level indices for the right-side of the distribution were computed in a similar way but using a sample for individuals who preferred Alternative 1 to Alternative 2 (i.e., x_i^r instead of x_i^l and n^r instead of n^l). For each country, the total number of individuals who are not indecisive between the two alternatives is shown in Tab. S3 while the number of left-sided and right-sided individual as a percentage of individuals who are not indecisive is shown in Tab. S7. The inequality symbols compare the created indices for the left-side and right-side of the distribution. A cell is shaded green if the difference between the left-side and right-side indices is greater than 3. Numbers are colored red if the difference between the left-side and right-side indices is greater than 10.

Survey Questionnaire

Start of Block: welcome

Welcome!

Thank you for participating in this survey. This is a research project on public acceptability of fisheries governance conducted by researchers from **Wageningen University** in the Netherlands. Answering all questions will approximately take **20 minutes**. If you have any questions about this project, please contact us at envecon@wur.nl. For more information, you can visit the website of Wageningen University.

End of Block: welcome

Start of Block: cheaptalk

The information obtained from this survey will give insights into the public acceptability of fisheries governance. Your name will NOT be used in data collection. Your answers will be treated in a confidential manner and your anonymity is guaranteed. There are no direct risks to you from taking part in this study. You can choose to decline to answer any question for any reason. You can also choose to remove yourself from participation at any time for any reason. However, we do hope you complete the survey in full.

The survey consists of 3 parts and at the beginning of each you will be given instructions about the type of decision that is required from you in each part.

End of Block: cheaptalk

Start of Block: consent

By participating in this study, it is important that you read the information below carefully and give your consent to participate in this study:

- You must be 18 years old or older.
- Researchers at Wageningen University and Research will use the information you provide in this survey. Information include both the choices that you make throughout this research and the answers that you give to survey questions.
- Your date will be used only for research purposes.
- Your data will be stored securely and encrypted in accordance with the guidelines of Wageningen University and Research.
- You understand that the information you provide will be anonymized and kept securely in accordance with the guidelines of Wageningen University and Research.
- You understand that it is possible that your data will be used for follow-up research, possibly by other researchers.
- You understand that participation is voluntary and that you are free to stop participating at any time, without having to give a reason. You can choose to withdraw your consent to participate even after you have completed the survey, by contacting us at envecon@wur.nl.

For more information, please follow this link (redirects to Wageningen University regulations for protection of personal data).

Please take a screen shot
to keep a copy of this consent form

By continuing with this survey, you confirm that you agree to the above statements.

End of Block: consent

Start of Block: part1

[Survey for another project]

End of Block: part1

Start of Block: part2

In this part of the survey, you will be asked to choose a spectrum between two options. As we have asked previously, please respond to each of the question below exactly as you would in real life. The results of this study may be made available to policy makers and could serve as a guide for future decisions.

End of Block: part2

Start of Block: dsi_text

Genetic resources can be utilized for various purposes, including but not limited to, medical research, drug development, and food production. These genetic resources come from animals, plants, and microorganisms. Genetic data can be stored digitally, which is known as Digital Sequencing Information (DSI). DSI provides a digital blueprint of an organism's genetic makeup, making it easier to conduct research and development activities without using physical genetic resources.

A major challenge remains on how to share the benefits from using DSI with the countries and communities in which the genetic resources originate from. In particular, developing countries, along with indigenous peoples and local communities, often lack access to technologies and resources that allow them to widely generate, access, use, analyse, and store DSI. As such, most of the benefits coming from the utilization of DSI remain mostly in developed countries.

The idea of a “benefit-sharing” mechanism of DSI means that when this information is used, the resulting benefits should be shared fairly across countries and communities. Those benefits may be monetary or non-monetary (i.e., exchange of knowledge, research infrastructure, and research collaborations). Benefit-sharing requires agreements between different stakeholders and governments, which may be difficult to achieve as everyone has different opinions on what is considered fair.

Currently, the benefit-sharing of (physical) genetic resources is arranged bilaterally – between the users (i.e., a research institution or a company) and the government of the country from which the genetic resources come from. It has been suggested that these bilateral agreements should be replaced by a multilateral mechanism. A multilateral benefit-sharing scheme could provide a structure in which different countries can collaborate and share benefits from DSI, while users wouldn’t need to approach individual governments to agree on benefit-sharing terms. A successful multilateral mechanism should establish a

framework for how benefits should be shared among all involved stakeholders and what form these benefits should take (i.e., monetary vs. non-monetary benefits). A key question is who should contribute

Page Break

While a multilateral benefit-sharing mechanism for DSI offers clear advantages, there is very little consensus on how this multilateral benefit-sharing should look like. The details of a multilateral benefit-sharing mechanism will be discussed at the 16th United Nations Biodiversity Conference of the Parties meeting (COP16), to be held this year. Several possible arrangements are currently being openly proposed and discussed.

Below, you will find some key elements that will determine how these arrangements will work in practice. For each pair of competing alternative elements on a benefit-sharing mechanism for DSI, you will be asked to allocate 100 points between these two alternatives. **Your final allocation between these two alternatives should sum up to 100**. The more points you allocate to an alternative, the more you agree with that alternative.

*

dsi_form What form should the benefits from the use of DSI take?
________ DSI benefits should primarily be monetary. (1)
________ DSI benefits should primarily be non-monetary. (2)

DSI benefits should primarily be monetary. The advantage of this alternative lies in the ease of use for both collection and distribution. The disadvantage of this alternative is on how it ignores other forms in which benefits could take place, such as capacity building in the countries where it is needed the most.

DSI benefits should primarily be non-monetary. The advantage of this alternative is the wide range of benefits that can take place. For instance, research collaborations, exchange and building of technical, technological, and human capacities. The disadvantage of this alternative lies in the difficulty of accounting for the share of non-monetary benefits.

Page Break

*

dsi_pay Who should pay for the utilization of DSI?
________ The users of DSI. (1)
________ The countries' governments. (2)

The users of DSI. Any private or public individual or organization who uses DSI should pay to the benefit-sharing multilateral mechanism. These users could either be individuals, research institutes, universities, or the industry. This alternative also allows for the differentiation among the users that should pay (e.g. depending on use of DSI or generation of money from DSI). The advantage of this alternative is that just the ones who use DSI are responsible for the payments. The disadvantage of this is the difficulty in tracing the users of DSI, which can lead to high avoidance of payments.

The countries' governments. The proposal is for governments to be responsible in directly paying to the multilateral mechanism. This payment can be done according to a specific criterion, such as in proportion to a country's economic wealth. The advantage of this alternative is its ease of implementation. That is, there will be no need to trace which individuals or organizations are using DSI, and the governments of these countries can decide how to fund these payments through taxes. The disadvantage of this alternative is on how economically wealthier countries can end up paying more even though they might be using DSI less. Moreover, if the government decides to create new taxes to pay for the use of DSI, it is unclear which stakeholders (i.e., companies, research institutions, or civil society) will end up paying these taxes.

Page Break

*

What should the money collected for the use of DSI be used for?
________ Countries' governments are free to allocate the money as they see fit. (1)
________ The money is earmarked for specific purposes. (2)

Countries' governments are free to allocate the money as they see fit. The advantage of this alternative is in the flexibility in which the money from the use of DSI is used. It could be used to support important non-DSI-related activities, such as education and child nutrition. The disadvantage of this alternative is that money coming from the use of DSI could be used for purposes that are not necessarily related with biodiversity, research and development in the life sciences, or social welfare.

The money is earmarked for specific purposes. DSI money is earmarked to support specific projects, such as those aimed at biodiversity conservation or research and innovation. Through such projects, the money could be distributed to specific countries. The advantage of this alternative is the clarity in which DSI money will be spent (e.g. on projects directly related to conservation and sustainable use of biodiversity). The disadvantage of this alternative is the rigidness of where the money will be spent and little autonomy for countries to decide themselves how to spend the money. Moreover, earmarking money for a specific purpose do not necessarily mean that the money is earmarked for projects that need it the most.

Page Break

*

How should the money from the use of DSI be distributed across countries?
_______ The DSI money should be equally distributed among countries. (1)
_______ The DSI money should be distributed among countries based on a specific criterion. (2)

The DSI money should be equally distributed among countries. This proposal implies that among the countries receiving benefits from the collected funds should get the money in equal shares. The advantage of this alternative is its ease of implementation. The disadvantage of this alternative is that countries might not consider this allocation fair, as the generation and use of DSI does not happen equally.

The DSI money should be distributed among countries based on a specific criterion. This proposal implies that countries receive the money according to some initially agreed upon criteria, such as the country's contribution to DSI data, the amount of biodiversity of the country, or the country's need for biodiversity conservation. The advantage of this alternative is on how countries that need the money more can get more money. The disadvantage of this alternative lies in the difficulty of creating and agreeing on such a criterion.

Page Break

End of Block: dsi_text

Start of Block: part3

In this part of the survey, you will be asked some general questions about yourself.

End of Block: part3

Start of Block: demog

What is your gender?

- ○ Male (1)
- ○ Female (2)
- ○ Other (3)
- ○ Prefer not to say (4)

What year were you born?
Years from 1950-2005 (1)

▼ 1950 (1) ... 2005 (56)

How many children do you have?

- ◯ 1 (1)
- ◯ 2 (2)
- ◯ 3 (3)
- ◯ 4 (4)
- ◯ 5 or more (5)
- ◯ I have no children (6)

Which country were you born in?
Countries (1)

▼ Afghanistan (1) ... Zimbabwe (195)

Which country do you currently live in?
Countries (1)

▼ Afghanistan (1) ... Zimbabwe (195)

How long have you lived in this country?

____

What is your residential postal code?

____

What is the highest educational level you have completed?

- ○ University (WO) or equivalent (2)
- ○ Applied university (HBO) or equvalent (3)
- ○ Vocational or equivalent (4)
- ○ None of the above (5)

Do you work in the fisheries sector?

- ○ Yes (1)
- ○ No (2)

What is your household's monthly net income in 2022?

- ○ 0 - 300 EUR (1)
- ○ 301 - 600 EUR (2)
- ○ 601 - 1000 EUR (3)
- ○ 1001 - 1200 EUR (4)
- ○ 1201 - 1700 EUR (6)
- ○ 1701 - 2500 EUR (7)
- ○ 2501 - 4000 EUR (8)
- ○ > 4000 EUR (9)

End of Block: demog

Start of Block: confidence

In general, how much confidence do you have in the government?

- Not a great deal (1)
- Not very much (2)
- Quite a lot (3)
- A great deal (4)

In general, how much confidence do you have in big companies?

- Not a great deal (1)
- Not very much (2)
- Quite a lot (3)
- A great deal (4)

In general, how much confidence do you have in environmental organisations?

- Not a great deal (1)
- Not very much (2)
- Quite a lot (3)
- A great deal (4)

In general, how much confidence do you have in new technologies?

- ◯ Not a great deal (1)
- ◯ Not very much (2)
- ◯ Quite a lot (3)
- ◯ A great deal (4)

Here are two statements people sometimes make when discussing the environment and economic growth. Which of them comes closer to your own point of view?

- ◯ Protecting the environment should be given priority, even if it causes slower economic growth and some loss of jobs. (1)
- ◯ Economic growth and creating jobs should be the top priority, even if the environment suffers to some extent. (2)

How much do you agree or disagree with this statement? "Many of the claims about environmental threats are exaggerated."

- ◯ Agree strongly (1)
- ◯ Agree (2)
- ◯ Neither agree nor disagree (3)
- ◯ Disagree (4)
- ◯ Disagree strongly (5)

Do you have any comments on the previous asked questions that you think are important/ can contribute to this survey?

____

End of Block: confidence
